# A Rare Inherited 15q11.2-q13.1 Interstitial Duplication with Maternal Somatic Mosaicism, Renal Carcinoma, and Autism

**DOI:** 10.3389/fgene.2016.00205

**Published:** 2016-11-25

**Authors:** Nora Urraca, Brian Potter, Rachel Hundley, Eniko K. Pivnick, Kathryn McVicar, Ronald L. Thibert, Christopher Ledbetter, Reed Chamberlain, Leticia Miravalle, Carissa L. Sirois, Stormy Chamberlain, Lawrence T. Reiter

**Affiliations:** ^1^Department of Neurology, University of Tennessee Health Science CenterMemphis, TN, USA; ^2^Pediatric Clinical Research Unit, Le Bonheur Children’s HospitalMemphis, TN, USA; ^3^Department of Pediatrics, University of Tennessee Health Science CenterMemphis, TN, USA; ^4^Neuroscience Institute, Le Bonheur Children’s HospitalMemphis, TN, USA; ^5^Division of Developmental Medicine, Department of Pediatrics, Vanderbilt University School of MedicineNashville, TN, USA; ^6^Department of Ophthalmology, University of Tennessee Health Science CenterMemphis, TN, USA; ^7^Department of Neurology, Massachusetts General HospitalBoston, MA, USA; ^8^Department of Urology, University of Tennessee Health Science CenterMemphis, TN, USA; ^9^Genetics Associates Inc., Nashville, TNUSA; ^10^Department of Genetics and Genome Sciences, University of Connecticut Health CenterFarmington, CT, USA

**Keywords:** 15q duplication, somatic mosaicism, renal carcinoma, stem cells, growth competition assay, autism

## Abstract

Chromosome 15q11-q13.1 duplication is a common copy number variant associated with autism spectrum disorder (ASD). Most cases are *de novo*, maternal in origin and fully penetrant for ASD. Here, we describe a unique family with an interstitial 15q11.2-q13.1 maternal duplication and the presence of somatic mosaicism in the mother. She is typically functioning, but formal autism testing showed mild ASD. She had several congenital anomalies, and she is the first 15q Duplication case reported in the literature to develop unilateral renal carcinoma. Her two affected children share some of these clinical characteristics, and have severe ASD. Several tissues in the mother, including blood, skin, a kidney tumor, and normal kidney margin tissues were studied for the presence of the 15q11-q13.1 duplication. We show the mother has somatic mosaicism for the duplication in several tissues to varying degrees. A growth competition assay in two types of stem cells from duplication 15q individuals was also performed. Our results suggest that the presence of this interstitial duplication 15q chromosome may confer a previously unknown growth advantage in this particular individual, but not in the general interstitial duplication 15q population.

## Introduction

**FIGURE 1:**
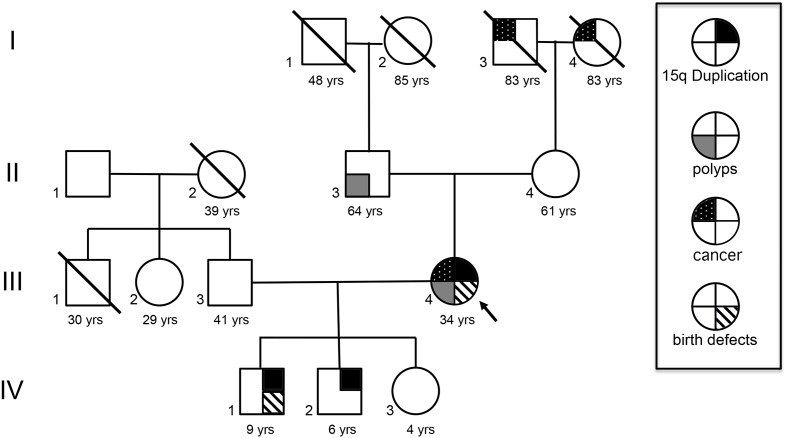
**Family history.** I-3 had prostate cancer and died at 83 years old. I-4 developed Kaposi sarcoma and died at 83 years old. II-3 had precancerous polyps and was negative for int dup15. III-4 was negative for int dup 15. The proband, III-4, had birth defects, renal carcinoma, and presented polyps like her father and passed the 15q duplication to her two sons, but not her daughter (IV-3). IV-1 has a maternal int dup15 and incomplete formation of the sacrum and coccyx and a pilonidal sinus tract. IV-2 with maternal int dup15.

A 35-year-old female underwent clinical and neuropsychiatric evaluation after her two affected sons, who have maternal interstitial 15q11.2-q13.1 duplication (int dup15) (**Figure 1**: IV-1 and IV-2) were seen at Le Bonheur Children’s Hospital. The mother was born with a unilateral preauricular pit. A sacral dermoid cyst, a large hemangioma on the left forearm, and another on the left inner thigh that were removed in childhood. She has a history of learning disabilities and attention deficit hyperactivity disorder. She graduated from high school and is currently a stay at home mom. At age 30, precancerous colonic polyps were removed. At 33 years of age she had a right subtotal nephrectomy due to renal carcinoma chromophobe cell type. A mild autism spectrum disorder (ASD) diagnosis was established at 34 year of age by the Autism Diagnostic Observation Schedule, Second Edition (ADOS-2) and Autism Diagnostic Interview, Revised (ADI-R) (Lord et al., 1989, 1994). She demonstrated an overall below average IQ of 75 on the Wechsler Abbreviated Scale of Intelligence second edition but there was significant discrepancy between reasoning domains with a low average Verbal Comprehension Index of 89 and well below average Perceptual Reasoning Index of 66.

She has a positive family history of cancer, including a father with similar precancerous polyps, and an 83-year-old maternal grandmother with Kaposi sarcoma, non-human immunodeficiency virus related. A maternal grandfather died at age 83 and had prostate cancer (**Figure 1**).

Fluorescence *in situ* hybridization (FISH) analysis was performed (Signature Genomics, Spokane, WA, USA) on blood and fibroblast cells. A probe specific for the 15q11.2-q13.1 region (*SNRPN* gene) along with two control probes targeting the chromosome 15 centromeric region (D15Z1) and the 15q22 region (*PML* gene) (Abbott Molecular, Abbott Park, IL, USA) were used to label metaphase cells and interphase nuclei. The results confirmed the presence of the 15q11.2-q13.1 duplication and demonstrated a somatic mosaicism, with 61.6% of blood cells and 67% of fibroblast cells containing the duplication (a total of 250 cells were assessed per sample).

**FIGURE 2:**
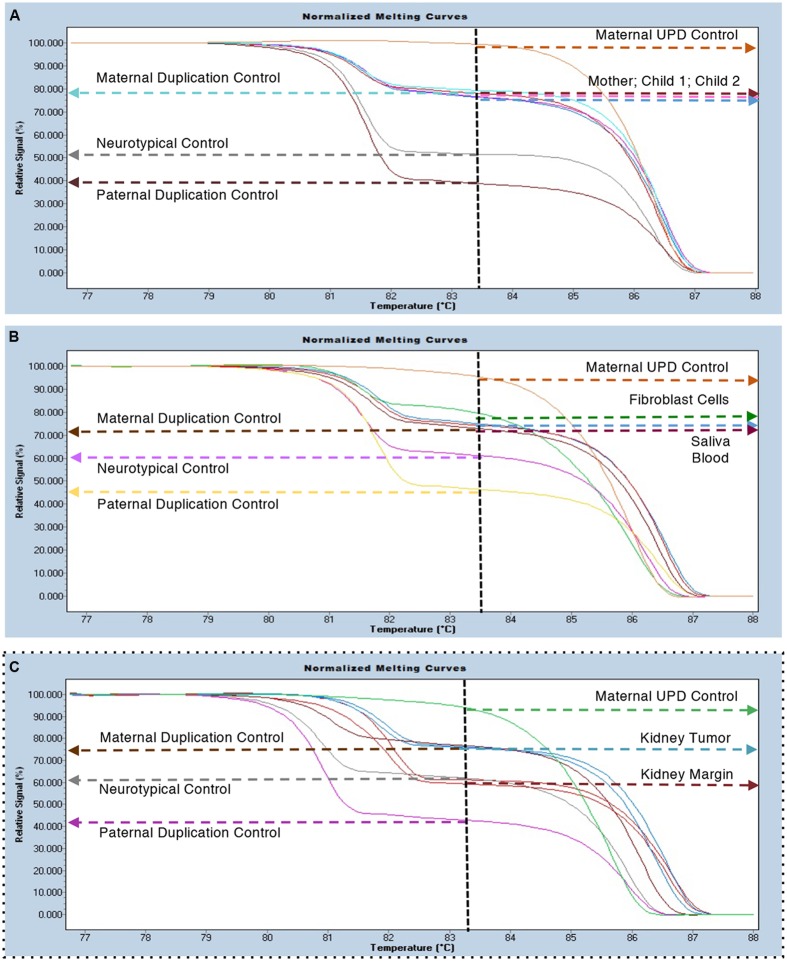
**MS-HRM duplication analysis. (A)** Blood samples from the proband (801-030) and her two affected sons (801-018 and 801-024) indicate the presence of a maternal specific duplication with an increased relative signal for the methylated (bisulfite converted) allele (i.e., 2:1). Maternal UPD (100% methylated); Neurotypical (55–65% methylated) and Paternal Duplication (35–45% methylated) controls are included for reference. **(B)** Survey of several tissues from the proband (801-030) including blood, saliva and a fibroblast cell line all show the presence of the maternally duplicated allele. **(C)** Samples from normal kidney margin co-localized with neurotypical control samples (*n* = 4) and samples from the tumor region co-localized with maternal duplication controls (*n* = 4).

Parent of origin testing by Methylation Sensitive-High Resolution Melting (MS-HRM) curve analysis from blood DNA of the differentially methylated region of the *SNRPN* gene (Urraca et al., 2010) indicated that this duplication is maternal in origin (**Figure 2A**). Her mild presentation suggested the need for formal testing in other tissues. Blood and saliva produced an identical signal to an interstitial maternal duplication control sample from blood (**Figure 2B**). However, her fibroblasts showed a slightly higher relative intensity than blood, reminiscent of isodicentric duplications (Urraca et al., 2010; Scoles et al., 2011) and possibly indicative of an increase in the number of copies of the *SNRPN* locus (**Figure 2B**). Renal tumor tissue samples were also analyzed. All tumor samples tested (*N* = 4) showed the presence of a maternal duplication, while the normal kidney margin samples from the same block (*N* = 4) showed a signal intensity that matched the control sample (**Figure 2C**). These results indicate that the faster growing tumor cells retained the interstitial duplication chromosome, while the surrounding normal tissues were predominantly non-duplication cells.

To determine if the int dup15 chromosome conferred a growth advantage in the renal carcinoma cells, we analyzed induced Pluripotent Stem Cells (iPSC) derived from her fibroblasts. We obtained 16 clones that had methylation profiles consistent with the presence of the duplication, and 2 clones with reduced methylation. Karyotype analysis indicated that one of the two clones with a normal karyotype was a mixed clone containing iPSC with the duplication and non-duplicated cells. The other clone appeared to be completely non-duplicated (20 cells analyzed). After repeated passage in the lab, the mixed iPSC culture was overtaken by the duplication cells. Repeated attempts to recover the iPSC with a normal karyotype from the mixed culture failed. We hypothesized that the non-duplication cells were being overgrown by duplication cells in this individual. To test this hypothesis, iPSC derived from an unrelated normal control individual were mixed with her duplication cell line, with a 1:1 ratio. In this mixed cultures, the unrelated normal karyotype iPSC were more abundant than the duplication iPSC compared to two different cell lines from the mother, indicating that the int dup15 does not confer a growth advantage in cultured iPSC (**Table 1**).

**Table 1:** Percentage of Dup15q Cells in Mixed induced Pluripotent Stem Cells (iPSC) and dental pulp stem cells (DPSC) Cultures.

Cell Line Combination	Cell Type	15q Duplication	15q Triplication
Control + Mother Line 1	iPSC	(38.5%) 77/200 cells	None
Control + Mother Line 2	iPSC	(11.2%) 15/132 cells	None
Control 1+ Dup15q 1	DPSC	(20.5%) 41/200 cells	(34.5%) 69/200 cells
Control 2+ Dup15q 2	DPSC	(6.5%) 13/200 cells	(13.5%) 27/200 cells
Control 3 + Dup15q 3	DPSC	(3.5%) 7/200 cells	(1.5%) 3/200 cells

**FIGURE 3:**
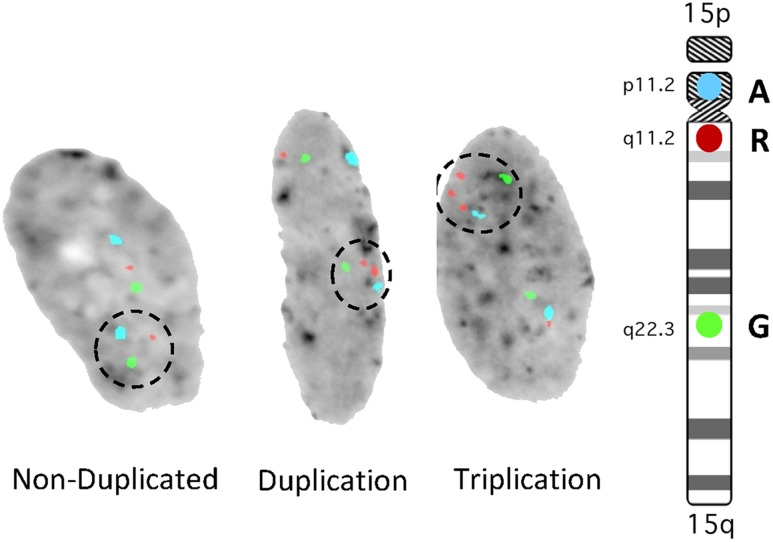
**Representative Fluorescence *in situ* hybridization (FISH) images from mixed culture analysis.** The aqua signal is probe D15Z1 located at the centromere, the red signal is a probe for the *SNRPN* gene within the duplication and the green signal is a probe for *PML* located outside of the duplicated region (internal control probe). Dashed circles indicate signals from a control chromosome, interstitial duplication and interstitial triplication chromosome.

To further evaluate the growth rate of non-duplicated vs duplicated cells in culture, Dental Pulp Stem Cells (DPSC) from unrelated controls and int dup15 cases were examined. Cells were mixed at 50:50 ratio and grown *in vitro*. The duplication cells in each of three experiments using six unrelated individuals consistently grew slower than the control cells (**Table 1**). In addition, we identified in each culture that 1–35% of the cells had undergone additional chromosomal rearrangements, resulting in triplication events (**Figure 3**). These results indicate that int dup15 cells typically grow slower than control cells in mixed culture from unrelated individuals and that the duplication is unstable in cultured DPSC.

### Son IV-1 (801-018)

Born prematurely at 34 weeks. At birth weight was 2.3 kg (60%) and 45 cm long (60%). He spent 5 days at the NICU for respiratory distress and required oxygen. He has a history of hypotonia and seizures from 6 weeks of age, now under control with oxcarbazepine therapy. Early motor and language developmental milestones were delayed. He receives special education as well as speech-language, occupational and physical therapy. Physical examination revealed at midline a flat vascular birthmark on the forehead. Other dysmorphic features include short palpebral fissures, long eyelashes, bushy eyebrows, mild droopy eyelids, and mildly lax facial features. He has hypotonia, a wide base to his gait and a left in toeing. Magnetic Resonance Imaging (MRI) of the brain was remarkable for periventricular leukomalacia. Lumbar spine MRI revealed an incomplete formation of the distal sacrum and coccyx and a tiny pilonidal sinus tract. Newborn abdominal ultrasound showed a prominent renal pelvis bilaterally.

The duplication status was determined by array comparative genomic hybridization (aCGH). The platform used was Genome-Wide Affymetrix SNP 6.0 array. The test confirmed the presence of a ∼5 Mb duplication encompassing the Prader-Willi/Angelman Syndrome (PWS/AS) critical region between BP2-BP3. At 5 years of age, he was administered the ADOS-2, Module 1. He had a Comparison Score of 10/10, suggesting a high level of ASD symptomology. He also met the diagnostic algorithm cut-off score on the ADI-R. Scores were consistent with clinical observations. On the abbreviated Stanford-Binet-V he demonstrated impaired intellectual ability with an IQ of 47.

### Son IV-2 (801-024)

Born full term with a weight of 3.8 kg (50%) and length of 52 cm (75%). At birth he presented with shoulder dystocia and difficulty breathing requiring intubation. He recovered and required no further treatment. Early motor and language development were thought to be normal. At 2 years of age, arrayCGH confirmed the same ∼5 Mb interstitial duplication found in his brother. Physical exam was positive for bilateral epicanthal folds, deep infraorbital creases and horizontal palpebral fissures, long eyelashes, short nose, flat nasal bridge, and a long philtrum. Lower extremity tone was decreased bilaterally, but in his upper extremities tone was increased and there was general joint laxity. He has near daily anger outbursts, during which he becomes physically aggressive, which is treated with risperidone. He attends a self-contained special education school and receives occupational, physical, and speech-language therapies. Brain MRI, echocardiogram and renal ultrasound were normal. At age 3.5 years of age he was administered the ADOS-2, Module 1 on which he had a Comparison Score of 8/10, suggesting a high level of autism-related symptomology. In addition, he met the diagnostic algorithm cut-off score on the ADI-R. Findings were consistent with clinical observations. On the Wechsler Preschool and Primary Scale of Intelligence (WPPSI-IV) he demonstrated below average IQ of 73; no discrepancy was noted between subdomains.

### Daughter IV-3

She was evaluated during her 1st year of life. No developmental, learning, or cognitive issues were reported. She was negative for any known copy number variants and appears neurotypical.

## Background

One of the most common copy number variants associated with ASD are duplications of the proximal arm of chromosome 15q11-q13.1 (Moreno-De-Luca et al., 2012; Al Ageeli et al., 2014) which can occur as either as interstitial, and more commonly, isodicentric duplications (Hogart et al., 2010). Widespread use of clinical microarrays has increased the detection rate of the smaller int dup15. Most individuals with int dup15 share the common deletion breakpoints of PWS/AS and result from a reciprocal non-allelic homologous recombination (NAHR) event. In a study of fourteen int dup15 cases, we reported that the phenotype includes mild facial anomalies, ASD, sleep issues, hypotonia, developmental delay, and a characteristic EEG variant (Urraca et al., 2013). We also confirmed in this cohort a previously reported parent origin effect (Cook et al., 1997; Schroer et al., 1998), while paternal duplication cases have incomplete penetrance (Urraca et al., 2013).

Most cases of int dup15 are *de novo* and maternally derived (Hogart et al., 2010). Given this maternal-specific effect and the low penetrance for ASD in paternal duplication cases, it has long been assumed that the few inherited int dup15 cases in the literature result from a silent (paternal) interstitial duplicated chromosome being passed on by the mother, resulting in a maternally inherited duplication in the offspring (Cook et al., 1997). Inherited int dup15 is rare, with only one published case involving the inheritance of a maternal duplication from the mother (Boyar et al., 2001), although some families with paternal duplication inherited from the father have been documented (Browne et al., 1997; Cook et al., 1997; Boyar et al., 2001; Veltman et al., 2005; Urraca et al., 2013; Al Ageeli et al., 2014). However, few cases of paternal duplication with developmental delay have been reported (Mohandas et al., 1999; Mao et al., 2000; Veltman et al., 2005).

## Discussion

This is an atypical family with a mother who is mosaic for maternal 15q duplication, with congenital anomalies, mild ASD, a history of unilateral renal cancer, and a positive family history for cancer. This maternal duplication was passed on to two affected children (IV-1 and IV-2). The anomalies and the renal cancer in the mother may not be related to the duplication, however, the two affected boys will be evaluated annually by renal ultrasounds. This family does not meet specific criteria for any familial tumor syndrome. Both affected boys presented with developmental delay, ASD and typical dysmorphic features previously described (Urraca et al., 2013), although IV-1 shares the sacral anomaly with his affected mother. The mother is only mildly affected cognitively, but still on the autism spectrum by clinical observation and formal ADOS/ADI-R testing. The fact that her FISH showed a significant decrease in the number of duplicated cells in both blood and skin raises the possibility that she may be mosaic for the duplication in the central nervous system (CNS) as well, thus decreasing the severity of her autism symptoms.

Here, we were able to evaluate both her normal and tumor renal samples and were surprised to find that the tumor cells consistently showed an int dup15 signal, while the surrounding normal kidney margin cells showed a typical control signal (**Figure 2C**). These results suggest that the faster growing tumor cells are those with the duplication, despite the documented somatic mosaicism. We tested the hypothesis that int dup15 cells grow faster than the non-duplicated cells in her iPSC using a growth competition assay. Interestingly, her duplicated cells grew faster than her normal cell line, however, when we performed further growth competition experiments using int dup15 iPSC and iPSC cells from non-duplicated unrelated donors, we found that the duplicated iPSC grew slower than the duplicated cells. Similar results were found when we examined the growth of cultured DPSC from six unrelated individuals (duplicated and non-duplicated) in a mixed culture assay, with duplicated DPSC cells growing slower than non-duplicated. It could be possible that the int dup15 confers a growth advantage just within this individual. These results imply that the 15q11.2-q13.1 duplication may not be the driving factor in the kidney tumor formation, but that some other event in these cells caused them to be the prominent cell type in the tumor cells versus normal kidney margin cells.

## Concluding Remarks

Here, we described a unique family with maternal int dup15 and a demonstrated somatic mosaicism in the mother. An extensive literature search also indicates that our proband is the first case of int dup15 with renal cancer, which appears coincidental since there is no apparent link between renal cell carcinoma chromophobe subtype and duplications of 15q (Ren et al., 2015). The mixed culture growth competition assays in two types of stem cells, iPSC and DPSC, also indicated that int dup15 cells typically grow slower than non-duplicated controls. However, these *in vitro* experiments may not accurately reflect the molecular and genetic events that occurred in the mother which resulted in tumor cells with the duplication and surrounding non-duplicated normal margin cells. Finally, the fact that the mother was mildly affected but did not display the full int dup15q phenotype implies that she may be mosaic for the duplication in her CNS as well as blood and skin.

## Ethics Statement

Informed consent was obtained and all experiments were performed in compliance with the University of Tennessee Health Science Center Institutional Review Board (IRB).

## Author Contributions

NU, EP, and LR conceived of the study, evaluated the data and wrote the manuscript; EP, BP, RH, RT, and KM performed clinical assessments and contributed to the manuscript; CL removed the kidney tumor and provided tissue; RC and CS performed experiments; LM and SC interpreted the data and contributed to the manuscript.

## Conflict of Interest Statement

The authors declare that the research was conducted in the absence of any commercial or financial relationships that could be construed as a potential conflict of interest.
